# The effects of climate warming on the migratory status of early summer populations of *Mythimna separata* (Walker) moths: A case‐study of enhanced corn damage in central‐northern China, 1980–2016

**DOI:** 10.1002/ece3.5739

**Published:** 2019-10-22

**Authors:** Qi Chen, Yun‐dong Zhang, Xiao‐hong Qi, Yong‐wei Xu, Yan‐hong Hou, Zhi‐ye Fan, Hai‐long Shen, Di Liu, Xing‐kai Shi, Shi‐min Li, Yun Duan, Yu‐qing Wu

**Affiliations:** ^1^ Luohe Institute of Agricultural Sciences Luohe China; ^2^ Key Laboratory of Integrated Pest Management on Crops in Southern Region of North China Zhengzhou China; ^3^ Henan Plant Protection and Plant Quarantine Station Zhengzhou China; ^4^ Institute of Plant Protection Henan Academy of Agricultural Sciences Zhengzhou China

**Keywords:** climate warming, migration, *Mythimna separata*

## Abstract

*Mythimna separata* (Walker) moths captured in light traps were monitored in Luohe, central‐northern China, from 1980 to 2016. Annual average temperature recorded an increase of 0.298°C/10 years in this region in the period. Our results indicate that a rising April and May average temperature and earlier occurrences of days recording the highest day temperature (30°C) caused an advanced peak and increasing proportion of high ovarian development levels of first‐generation females in earlier summers. Results using Johnson's formulation of “oogenesis‐flight syndrome” indicate that increasing sexual maturity proportion has resulted in more emigrant individuals in the local first‐generation moth becoming residents, and then increased individuals rapidly in the local second‐generation moth since 2006. Consequences of this action have a boom in corn damage since 2007 in this region. Advanced peak dates of the first and second‐generation moth revealed the same response to increasing average monthly temperatures in the monitoring period. Increasing temperatures, the average May temperature exceeds or equal to 22°C, during the early 2000's may represent a physiological threshold for *M. separata* development. Our results suggest that climate warming may impact *M. separata* migratory status and cause a problem of crop production in this region.

## Graphical abstract

*Mythimna separata* (Walker), an important migratory insect pest, were monitored in Luohe, central‐northern China, from 1980 to 2016. With the data of monitoring and ovary dissection, we found more emigrant individuals becoming residents and enhance corn damage in this region over this period because earlier occurrences of days recording the highest day temperature (30°C). Climate warming may impact *M. separata* migratory/residence status in its early summer population and cause a problem of summer‐fall corn production in the northern central China.

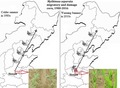

## 1 INTRODUCTION

The oriental armyworm, *Mythimna separata* (Walker), is a seasonal migratory insect pest that frequently causes serious widespread loss or even disasters in cereal crops in China and East Asia (Li, Wang, & Hu, 1964). Previous studies have reported that *M. separata* has four seasonal migrations in multigeneration between southern overwintering areas and northern areas every year in China. These migration episodes include two northern events (in spring and early summer) and two southern events (in the fall; Chen, Sun, Wang, Zhai, & Bao, 1995; Li et al., 1964; Wu, Cheng, Xu, Zhai, & Guo, 2001). The first northward migration occurs during the spring, ranging from southern areas to the central‐northern plain. During this episode, individuals colonize and damage wheat. The second northward migration occurs in early summer, ranging from central‐northern China to north and northeastern China. Generation individuals in this migration rarely remain and damage summer corn in the migration area, the central‐northern region (Feng et al., 2008; Li et al., 1964; Zhao et al., 2009). Recent reports, however, have recorded *M. separata* to increasingly cause destruction on a large scale on corn, the main grain crop, in summer and fall over recent decades in central‐northern China (Jiang, Zhang, Cheng, & Luo, 2014; Jiang, Li, Zeng, & Liu, 2014). Changes in the summer migratory status of *M. separata* should be evaluated for their impact on human activities and environmental change.

The occurrence of insect migration is closely connected to their physiological and ecological factors, such as habitats, feeding, and reproduction by modifiable environmental factors (Dingle, 1972; Jiang, Luo, Zhang, Sappington, & Hu, 2011). For example, the reduction of wheat area in southern China has resulted in a reduction of host plants (both food and habitat), an area where *M. separata* overwinters, thus resulting in a decrease of individuals migrating from the southern region to the central‐northern area. Due to these changes, wheat disasters related to *M. separata* during the 1980s and 1990s reduced in Guo's paper (2006). However, on the other hand, did enhance global warming result in an increase of *M. separata* and therefore an increase in crop damage in central‐northern areas? Surface mean temperatures in north China have been recorded to have increased by 0.22°C/10 years from 1960 to 2009 (in *China's second national assessment report on climate change*). Recently, studies have shown that migratory insects/birds have a response to climate change (Diffenbaugh, Krupke, White, & Alexander, 2008; Garcia, Godoy, Thomas, Nagoshi, & Meagher, 2017; Inouye, Barr, Armitage, & Inouye, 2000; Sparks, Dennis, Croxton, & Cade, 2007; Sparks, Roy, & Dennis, 2005; van Gils et al., 2016).

With the formulation of “oogenesis‐flight syndrome,” or “incompatible” physiological states of reproduction and migration (Johnson, 1963), it was shown that the migratory status of insects could be altered if rising temperatures changed reproductive development or sexual maturity. Results by Wang and Liu (1996) showed that an increase in environmental temperature (to between 21 and 27°C) could stimulate reproduction development and the release of *M. separata* sex pheromones. Cusson, Mcneil, and Tobe (1990) also showed that when *M*. (*Pesudaletia*) *unipuncta* adults were transferred from an environment with a temperature of 15 to one of 25°C, a significant increase in JH biosynthesis was recorded within 24 hr.

As an indicator of migratory status (Johnson, 1963), reproductive development levels of *M. separata* moths driven by temperature may be merely associated with climate warming through long‐term monitoring. Although recent studies have developed novel methods to investigate migration based on morphological characteristics (Zhang, Jiang, & Luo, 2008), using within‐wing isotopic technology (Hobson, Doward, Kardynal, & McNeil, 2018) and speculation of physiological, environmental, and genetic regulation (Jiang et al., 2011).

To understand the incidence of *M. separata* on corn in China during recent summers, we carry on environmental factors, such as climate change, are related to population dynamics and ovarian development of *M. separata* moths, with monitoring data in Luohe City, central China, during 1980 and 2016.

## 2 MATERIALS AND METHODS

### 2.1 Study site

The monitoring site was located in Luohe City, Henan Province, China (33°58′N, 113°99′E). This site is situated further north than the boundary edge (33°N) of where *M. separata* is thought not to be able to overwinter.

### 2.2 Collection method

Moths were collected between April 1st and October 31st, 1980 to 2016 (the 2004 record is missing), using light traps, which were located in the agricultural field of our institute's farm (33°36′N, 113°59′E). The black light wavelength used for collection was 365 nm with a power of 20 watts. Male and female individuals that were captured were counted every night.

### 2.3 Ovarian dissection

We randomly dissected the ovaries of 20 females if there were more than 20 every day, or of all females that were captured on a given night if the total number of females was <20. The proportion of mated females were checked, and the number of perm beads (represented mating times) in the female spermathecae were counted. Ovary data were used to determine the ovarian development level and mating rates. Moths were not dissected between 2005 and 2010, and in 2012. The ovarian development levels were divided into five grades as described by Dai, Jiao, and Qian (1962).

Grade 1: The ovarioles are transparent, fine (like a thread), and oocytes do not develop. These ovaries have no yolk deposition or enlargement of the oocytes. The ovarioles have not yet colored and appear milk white.
Grade 2: Oocytes near the oviduct appear yellow by yolk deposition. The ovaries are full and they do not contain mature eggs.
Grade 3: Fully mature eggs are observed in the ovariole and in the oviduct on mated females.
Grade 4: Nonfully mature eggs are present in the ovarioles and oviduct.
Grade 5: Few mature eggs are present in the ovarioles and oviduct, with ovarioles containing wastage.

With the hypothesis that emigration is generally initiated by sexually immature individuals, leading to the formulation of “oogenesis‐flight syndrome” (Johnson, 1963), local female moths were considered as emigrants (Lin, 1990) when ovarian development was categorized as grade 1 or 2. In these grades, female moths were virgins and sexually immature (Zhao et al., 2009), but residents when sexually maturity above grade 3 after they had mated.

### 2.4 Meteorological, corn planting, and *M. separata* damaged area data

Meteorological data were collected at local meteorological stations in Luohe city for the period spanning 1980–2016. Corn growth area and insecticide application area data since 1980 for *M. separata* were provided by the Henan Province Plant Protection and Plant Quarantine Station.

### 2.5 Data analysis

According to the seasonal migratory pattern of *M. separata* using Li's report in central China (1964) and our observation, there are four generations and moth peak periods with light trapped *M. separata* in Luohe area, central China (Figure S2).

Generations are divided by the date of valley bottom between two adjacent generation peak periods.

1. The immigrated moth population in spring from south China (spring arrivals): April to early May.
2. First local generation moth population occurs in early summer: mid‐May to late June.
3. Second local generation moth population occurs in late summer and early autumn: late June to early August (2000's) or mid‐August (1980's).
4. Third generation moth population occurs in autumn: late‐mid August to October.

In order to examine population dynamics, changes of phenological periods and migratory status correlating with climate warming, indices of light trapped individuals of every generation were extracted and converted by logarithm in Figure S2. The first emergence dates of spring arrival populations were recorded. Peak dates of the first and second generations were recorded, as well as the sexually mature proportions with females above ovarian development grade 3, mating proportions, and annual and monthly average temperatures. Dates of occurrence for the highest day temperature (30°C) between mid‐May and mid‐June were also recorded.

## 3 RESULTS

### 3.1 Monitoring number and emergence date changes of light trapped *M. separata* moths

**Figure 1:**
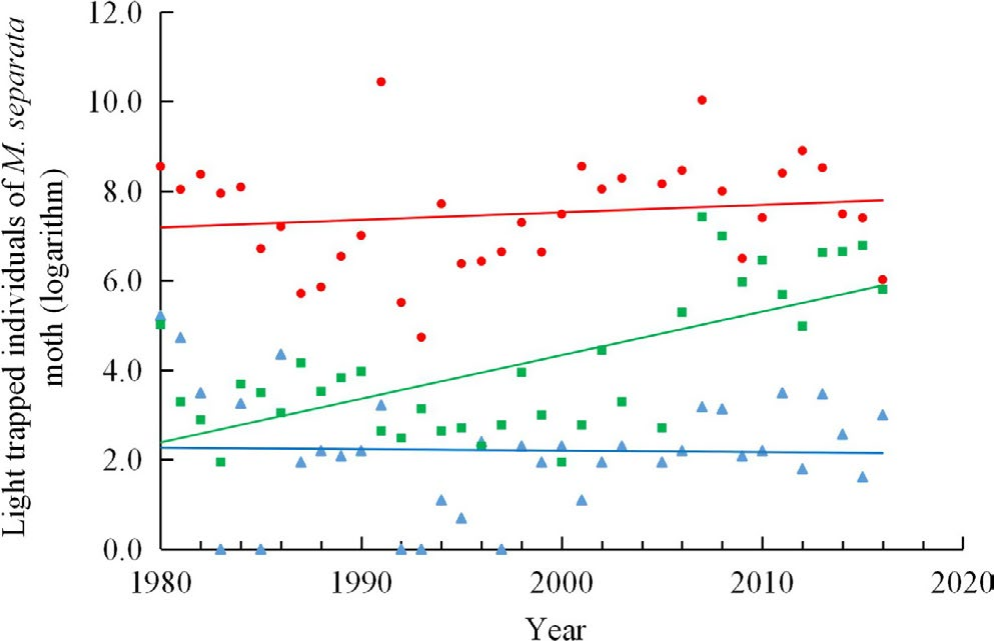
Individuals of light trapped *Mythimna separata* moths from 1980 to 2016. Triangles represent spring arrival population, circles represent the first generation, and blocks represent the second generation

Numbers of light trapped *M. separata* moths did not record a significant increasing or decreasing trend in the spring arrival/immigrated and the local first‐generation moths from 1980 to 2016, but a significant increase in the second generation in later summer was recorded (Figure 1; Spearman's correlation, the spring arrival moth, *n* = 34, *r* = −.182, *p* = .428, no moths were captured in 1983 or 1985); the first‐generation moth, *n* = 36, *r* = .157, *p* = .361; the second‐generation moth, *n* = 36, *r* = .544, *p* = .001). However, there is a discontinuity at about year 2006, after which the second‐generation numbers appear to jump to a higher baseline. The trap number/year is 222.81 ± 63.48 (*SE*) after 2006, but 33.83 ± 6.77 (*SE*) between 1980 and 2005. The second‐generation data in two periods, before 2005 and after 2006, didn't show linear trends. At the same time, *M. separata* damage corn areas in autumn have increased dramatically in this region since 2006 (Figure S3) too.

**Figure 2:**
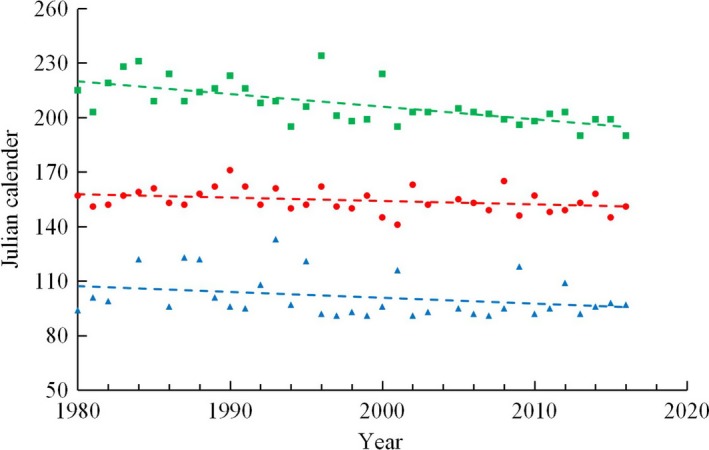
The earliest arrival dates of spring arrival *Mythimna separata* population (blue triangle) and advanced peak dates of the first (red circle) and the second generation (green block)

Results during the monitoring period showed a significant advance of emergence dates of light trapped *M. separata* moths (Figure 2; Figure S2). The earliest arrival dates were significantly earlier (by 3.21 days per 10 years) in the spring arrival population (*y* = −0.3213*x* + 743.38, Spearman's correlation, *n* = 34, *r* = −.323, *p* = .062). The light trapped moth peak dates of the first‐ and second‐generation advanced by 1.87 (*n* = 36, *r* = −.334, *p* = .047) and 4.44 (*n* = 36, *r* = −.709, *p* = .000) days per 10 years, respectively.

### 3.2 Results of *M. separata* females ovarian dissection

Ovarian dissection of 207 overwintering generations, 3,738 first‐generation and 1,386 second‐generation females were undertaken between 1980 and 2016. The ovarian development grades of annual dissected individuals were above three for all of spring arrived females. This result indicates that these individuals belong to exotic immigrated individuals which reached sexual maturity in the spring.

**Figure 3:**
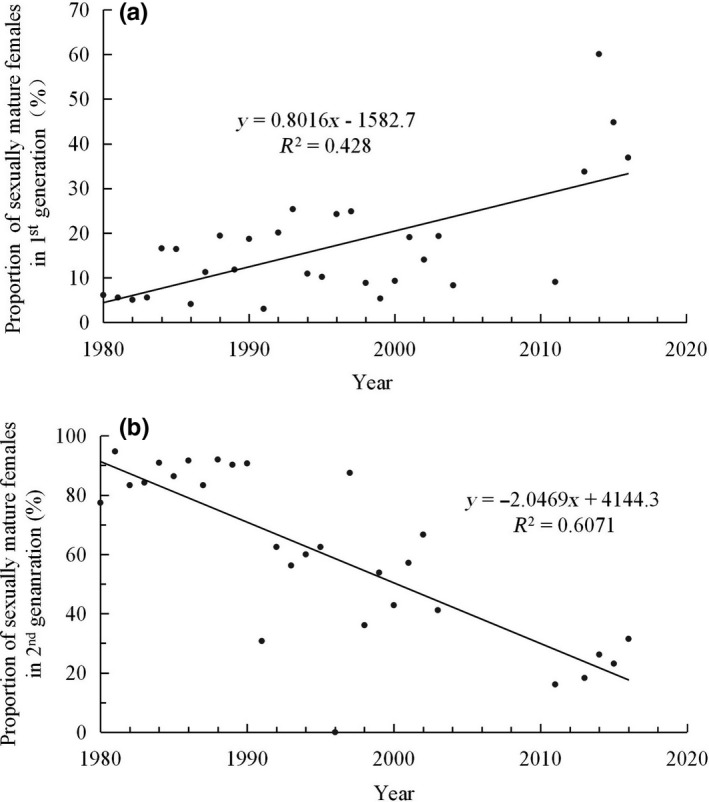
Proportions of sexually mature females in the first (a) and the second (b) generation moths during the monitoring period

The proportion of ovarian development of mature females (Figure 3a: Spearman's correlation, *n* = 30, *r* = −.508, *p* = .004) in first‐generation recorded a significant annual increase in this period. Proportions of mature ovarian development were <20% in the 1980s, but more than 30% after 2013. Mated proportion has the same trend.

Adversely, the proportion of the second generation in late summer recorded a significant decrease during the monitoring period. This result, therefore, means that more and more moths will immigrated to other area in late summer (Figure 3b).

**Figure 4:**
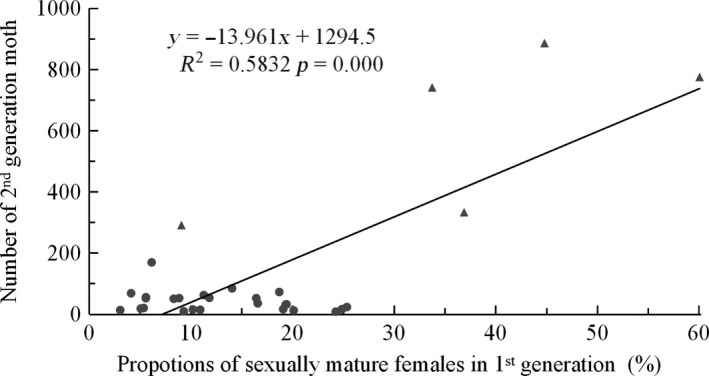
Increasing numbers of the second‐generation moths with increasing proportions of sexually mature females in the first generation during the monitoring period. Triangles represent data after 2006

An increase of the number of the second‐generation moths did not have a correlate with the first‐generation (Pearson correlation, *n* = 36, *r* = −.181, *p* = .292), but it has a close connection with increased proportions of sexually mature females (Pearson correlation, *n* = 29, *r* = .639, *p* = .000; Figure 4). This result means that the second‐generation moth is from residents of the first generation due to more sexually mature individuals in the first generation using Johnson's hypothesis (1963), especially there were merely fewer individuals (Figure 1 & plus 2) or very late occurrence (Figure 2) of the second‐generation moths before 2005 in our observation site.

### 3.3 Phenological responses to climate warming

**Figure 5:**
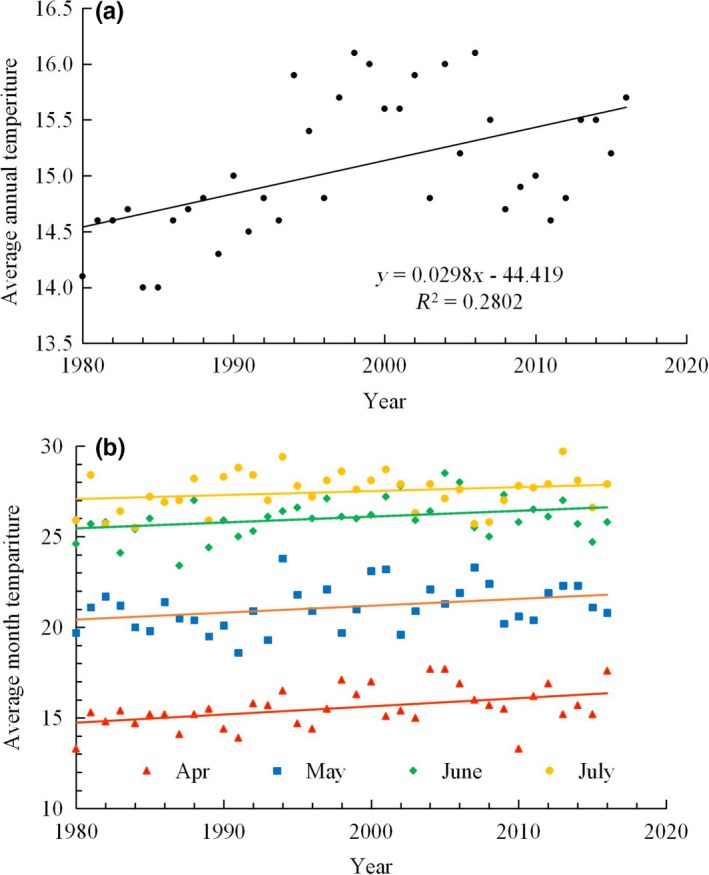
Warming trends in Luohe city, 1980–2016. a is the average yearly temperature, b is the average monthly temperature

Annual average temperature recorded an increase of 0.298°C/10 years in the Luohe monitoring site from 1980 to 2016 (coefficient of determination *R* ^2^ = .260, *F*_1,35_ = 13.624, *p* = .001; Figure 5). From April to June, average monthly temperatures significantly increased during the period coinciding with the occurrence of *M. separata* (April *R* ^2^ = .1921, *F*_1,35_ = 8.332, *p* = .007; May *R* ^2^ = .113, *F* = 4.471, *p* = .042; June *R* ^2^ = .150, *F* = 4.124, *p* = .050). Although average July temperatures also recorded an increasing trend, this was not significant (*R* ^2^ = .050, *F* = 1.825, *p* = .185).

**Figure 6:**
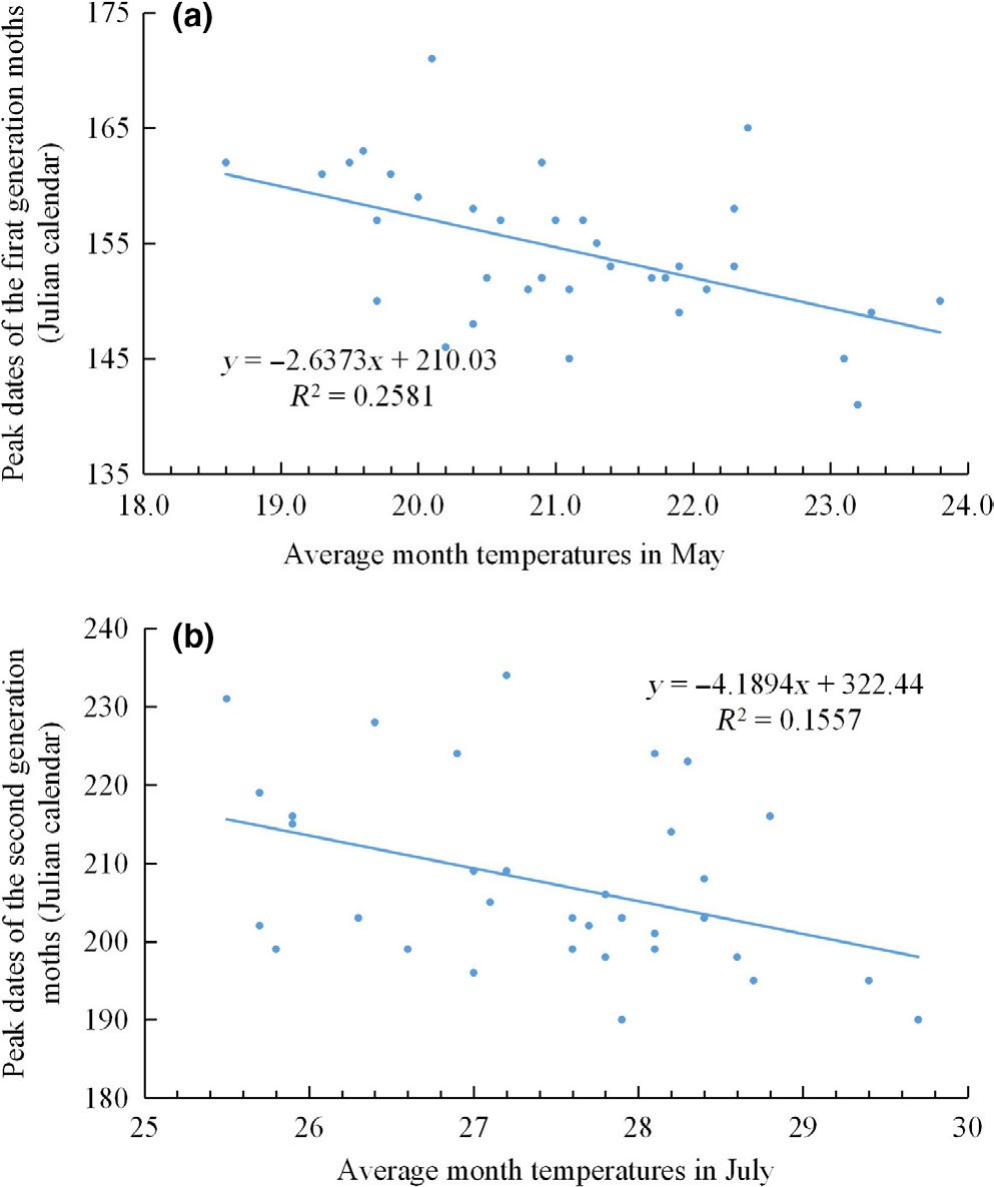
Peak dates of *Mythimna separata* moth response to increasing average monthly temperature. a is the first‐generation population in early summer, b is the second‐generation population in late summer

Dates of first capture of the spring arrivals did not show a relationship between progressively earlier days and average April temperatures (*R* ^2^ = .021, *F*_1,33_ = 0.694, *p* = .411; 1983 and 1985 had no captured individuals in spring). However, advanced moth peak dates of the first‐ and the second‐generation *M. separata* were closely related to increasing average temperatures in May and July in this period, respectively (first generation: *R* ^2^ = .258, *F*_1,35_ = 11.829, *p* = .002; second generation: *R* ^2^ = .156, *F*_1,35_ = 11.6272, *p* = .017, Figure 6). These results clearly show that *M. separata* has a strong response to increasing temperatures.

### 3.4 High temperature in summer promoted sexually maturity of the first generation of *M. separata* female moths

**Figure 7:**
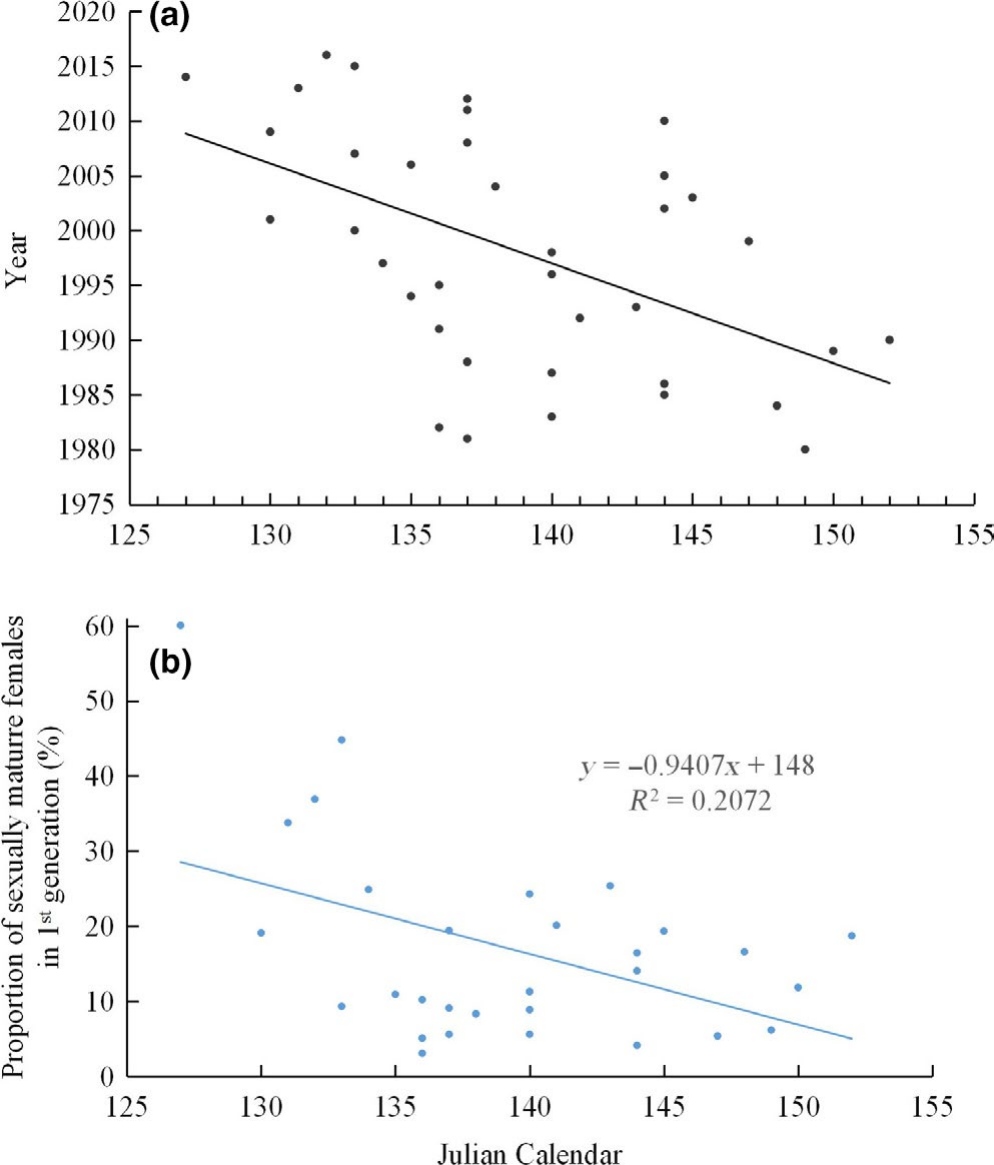
Climate warming impact on migrated status (reproductive development). a is the date when the highest day temperature is 30°C between Mid‐May and Mid‐June; b is the rates of sexually immature female in the first‐generation *Mythimna separata* moth's response to climate warming in the monitoring period, respectively

Climate warming has resulted in high temperatures date becoming progressively warmer earlier (van Gils et al., 2016). During the emergence period of the first‐generation moth, we found that the occurrence date when daily temperature attained 30°C in May (early summer) had advanced at a rate of 0.911 days per year (*b* = −0.9115, *R* ^2^ = .266, *F*_1,35_ = 12.700, *p* = .001, Figure 7a). As advancing date attained 30°C in May, there were increasing proportions of sexually mature females in the first generation (*R* ^2^ = .207, *F*_1,29_ = 7.318, *p* = .011, Figure 7b). This result means that high temperature promotes sexual maturity of the first‐generation moths, and its more individuals become residents (Figure 4) with Johnson's formulation of “oogenesis‐flight syndrome” Johnson, 1963).

## 4 DISCUSSION

In this paper, we show an important aftermath that phenological changes of *M. separata* had a strong response to a warming environment, similar to other species (Bebber, Ramotowski, & Gurr, 2013; Ovaskainen et al., 2013), including an increase of sexually mature females, and changes of its migratory and damage status.

Known responses to climate change on migratory species include (a) progressive arrival in the emigratory region (Sparks et al., 2007; Sparks et al., 2005); (b) expanded survival or crop damage margin (Diffenbaugh et al., 2008; Garcia et al., 2017); and (c) a reduction in body size due to malnutrition (van Gils et al., 2016). However, physiological changes were not reported for the altered migratory status of species due to climate warming.

The promotion of sexual maturity of *M. separata* female moths with an increase in temperature has been previously recorded in laboratory by Cusson et al. (1990), Heinrich (1993) and Wang and Liu (1996). Results from our study support the findings that the migratory status of early summer populations of *M. separata* moths changed since the 1980s on the central‐northern China plain as early summer warming accelerated ovarian development.

Previous studies have shown that early summer population emigration occurred from central‐northern China to north and northeastern China (Feng et al., 2008; Li et al., 1964; Zhao et al., 2009), and almost all individuals emigrated. However, the emigratory proportion was reduced due to global warming in recent decades, coupled with a decreasing proportion of lower ovarian development of *M. separata* female moths by the formulation of “oogenesis‐flight syndrome” (Han & Gatehouse, 1991; Johnson, 1963, 1969). Matured ovarian individuals would remain in central‐northern China, leading to an increase of next‐generation individuals in late summer, thereby this could result in an increase in corn damage.

With official data of the Henan Plant Protection and Plant Quarantine Station, there was a boom in the area of damaged corn since 1980 in this region (Figure S3). In the 1980s, the area of damaged corn from June to August was <80 thousand ha, but it was over 400 thousand ha from 2007 to 2016. There were three breaks in 2011, 2014, and 2015 in this region. At the same time, there was a rapid growth of the area of insecticide application for *M. separata* control in the corn.

A bloomed increase of individuals of the second‐generation moths and corn damaged area emerged are almost simultaneous in 2007. So this fact supports that more local first‐generation individuals of *M. separata* in early summer remained and injured corn in next season in this region.

Although results mean why is an increase in the area of damaged summer corn and individuals of the second‐generation moths in late summer from climate warming in this region, we wonder if “a threshold” of climate warming for a change of *M. separata* migratory status began in 2006. Jiang et al. (2011) described a sensitive period in early adulthood where specific environmental factors can inhibit *M. separata* migratory behaviors, most flight occurs at temperatures between 11 and 32°C, with an optimum of ≈17–22°C. There were only 2 years when the average May temperature exceeds or equal to 22°C during 1980 and 1999, but 9 years from 2000 to 2018 (Figure 5b). Increasing temperatures during the early 2000's may represent a physiological threshold for *M. separata* development.

On the other hand, insect migration behavior is also triggered or inhibited by environmental factors, such as habitat quality influenced by host plants except climate factors (Ramenofsky & Wingfield, 2007). In our study, as host plants, the stable planting of winter wheat science 1980 (Wang, Tian, Guo, & Wang, 2009), the only factor to have changed may be climatic effects.

## 5 CONCLUSION

Our conclusion suggests that a warming climate in early summer could change migrant individuals of *M. separata* to being resident by an increase of female sexually mature between 1980 and 2016. This would result in them frequently occurring on corn in the summer in the central‐northern area in China (Figure S3; Jiang, Zhang, et al., 2014; Jiang, Li, et al., 2014; Li, 1994; Sujayanand & Karuppaiah, 2016).

## CONFLICT OF INTEREST

None declared.

## AUTHOR CONTRIBUTIONS

Q. C. and Y. W. designed the work, drafted the manuscript, and revised it critically for important intellectual content. Y. Z. and S. L. participated in drafting the manuscript too and were responsible for the data management as main managers. Q. C., X. Q., Y. X., Z. F., Y. H., H. S., D. L., and X. S. made substantial contributions to the acquisition of data for the work. Y. D. has made analysis of data partially. All authors reviewed the manuscript.

## Data Availability

The moth monitoring and meteorological data in Luohe City were archived in Dryad upon acceptance, https://doi.org/10.5061/dryad.f9r12h3.
