# Supplementary figures and images for: The effects of climate warming on the migratory status of early summer populations of Mythimna separata (Walker) moths: A case‐study of enhanced corn damage in central‐northern China, 1980–2016
Source: Ecol Evol. 2019 Oct 22;9(21):12332–8. doi: 10.1002/ece3.5739 (PMC6854107; doi:10.1002/ece3.5739)

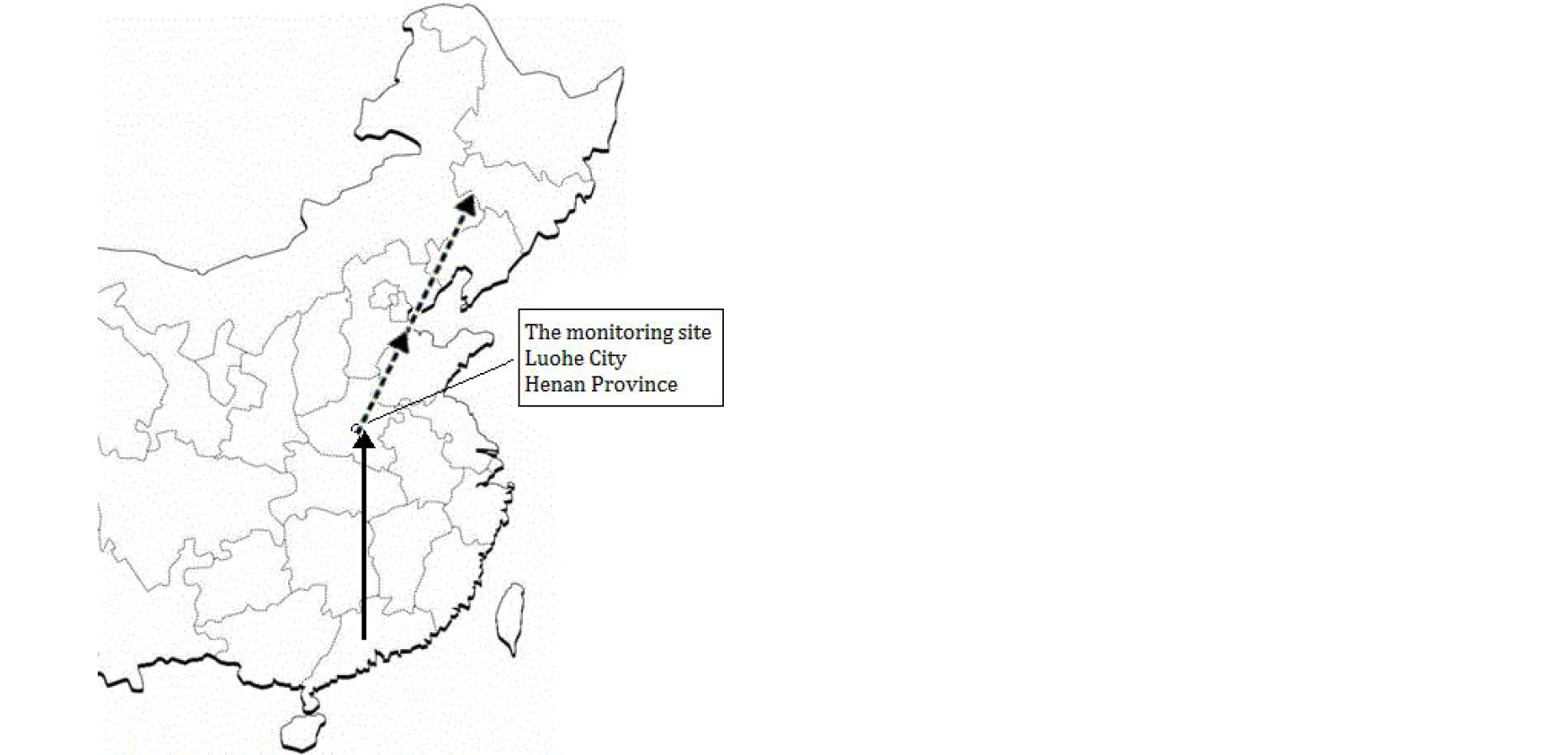

Supplement: Supplementary file 1 [file ECE3-9-12332-s001.tif]

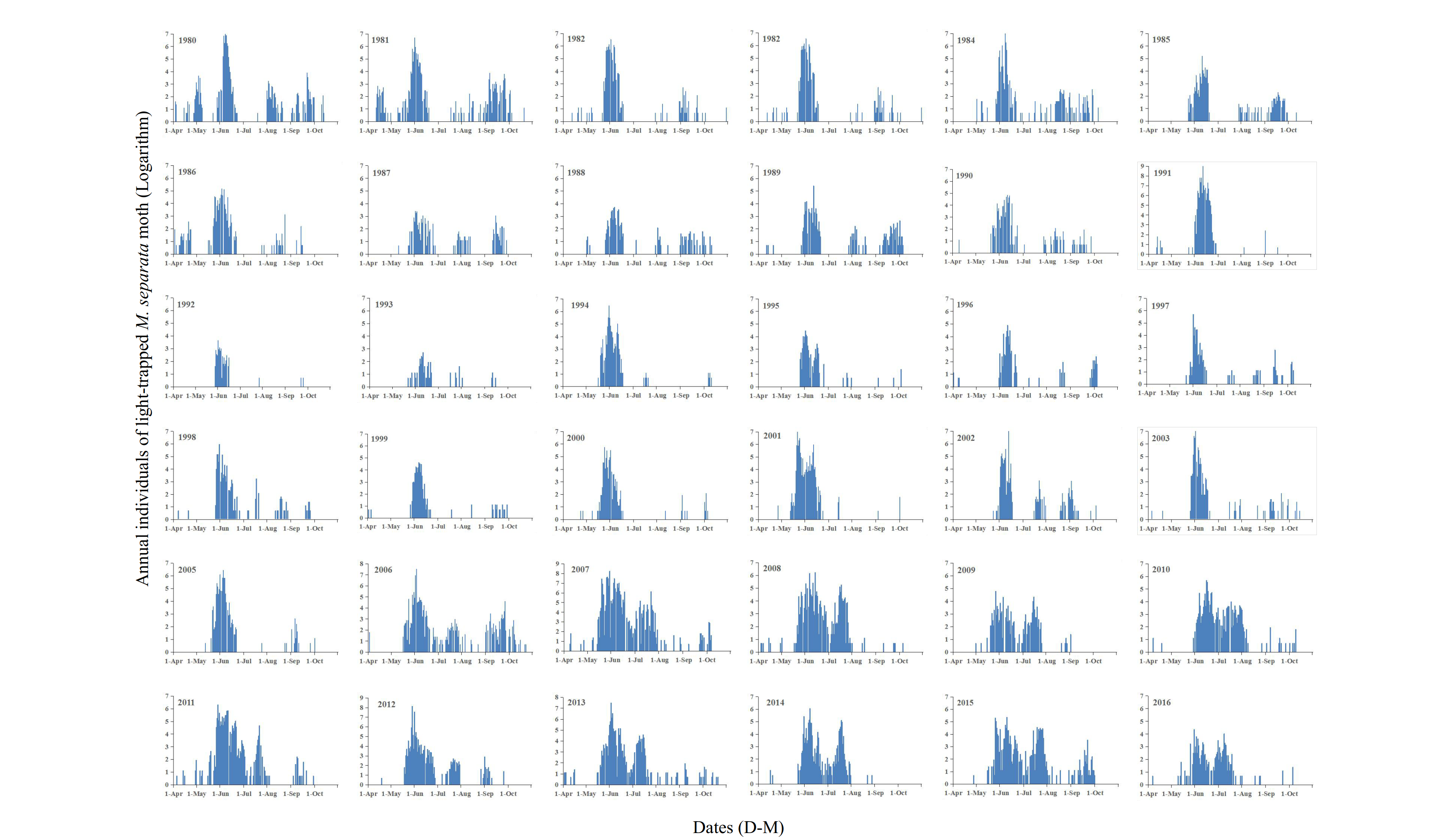

Supplement: Supplementary file 2 [file ECE3-9-12332-s002.tif]

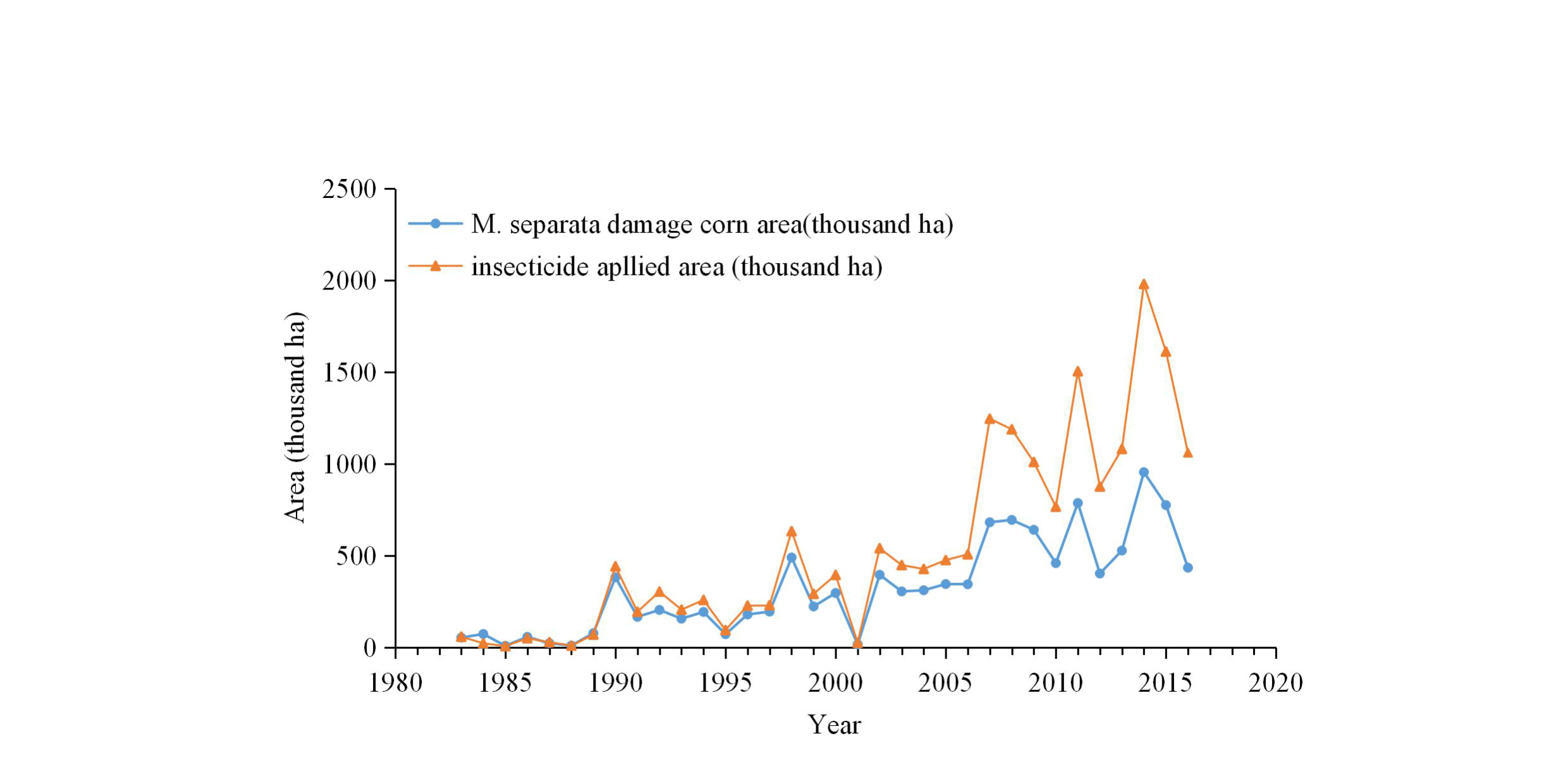

Supplement: Supplementary file 3 [file ECE3-9-12332-s003.tif]
