# Supplementary material for: The effects of climate warming on the migratory status of early summer populations of Mythimna separata (Walker) moths: A case‐study of enhanced corn damage in central‐northern China, 1980–2016
Source: Ecol Evol. 2019 Oct 22;9(21):12332–8. doi: 10.1002/ece3.5739 (PMC6854107; doi:10.1002/ece3.5739)
Supplement: Supplementary file 4 [file ECE3-9-12332-s004.docx]

Figure S1. Map shows the position of the Luohe City, Henan Province, China mainland, the monitoring site, and possible *M. separata* migration trajectories. Dashed arrows, trajectory of spring migration of the overing winter generation, and black arrows, direction of early summer migration of the first generation. shown by previous studies (Li et al., 1964).

Fig S2. Annual population dynamics of light-trapped *M. separata* moth from 1980 to 2016.

Fig S3 Increasing *M. separata* damage corn area and insecticide applied area in Henan, North Central China
